# Kin-Avoidance in Cannibalistic Homicide

**DOI:** 10.3389/fpsyg.2020.02161

**Published:** 2020-08-31

**Authors:** Marlies Oostland, Michael Brecht

**Affiliations:** ^1^ Bernstein Center for Computational Neuroscience Berlin, Humboldt-Universität zu Berlin, Berlin, Germany; ^2^ Princeton Neuroscience Institute, Princeton University, Princeton, NJ, United States

**Keywords:** cannibalism, kin, homicide, evolution, mental health

## Abstract

Cannibalism in the animal kingdom is widespread and well characterized, whereas the occurrence of human cannibalism has been controversial. Evidence points to cannibalism in aboriginal societies, prehistory, and the closely related chimpanzees. We assembled a non-comprehensive list (121 offenders, ~631 victims) of cannibalistic homicides in modern societies (since 1900) through internet-searches, publications, and expert questioning. Cannibalistic homicides were exceedingly rare, and often sex-related. Cannibalistic offenders were mainly men and older than offenders of non-cannibalistic homicides, whereas victims were comparatively young. Cannibalistic offenders typically killed manually (stabbing, strangulating, and beating) rather than using a gun. Furthermore, they killed more strangers and fewer intimates than conventional offenders. Human cannibals, similar to cannibalism in other species, killed and ate conspecifics, occasionally vomited and only rarely (2.5% of victims) ate kin. Interestingly, cannibalistic offenders who killed their blood relatives had more severe mental problems than non-kin-cannibals. We conclude that cannibalistic homicides have a unique pattern of murder methods, offenders, and victims.

## Introduction

Greek mythology is filled with examples of rape, incest, and bestiality, but only an act of familial cannibalism is truly beyond the pale. The god Cronus, having learned of a prophecy that he would be usurped by his son, devoured his own children as soon as they were born. His wife Rhea managed to save only Zeus, the sixth child. When Zeus became an adult he faced his father, forced him to return the swallowed children, and ousted him from the throne, becoming the supreme lord of all the gods. To this day, the subject of human cannibalism remains contentious. For instance, anthropology experienced a heated debate on the existence of aboriginal cannibalism (Kolata, 1986; Barker et al., 1998; Sahlins, 2003; Arens, 2004).

Cannibalistic homicides, acts in which a human is killed and parts of the body are consumed or prepared for consumption, are one of the most extreme variants of homicide offenses. Examples of extreme variants of non-cannibalistic homicide offenses are serial homicides and sexual homicides (Chan et al., 2010; Beauregard and DeLisi, 2018), in which offenders often have psychopathic features (Boduszek et al., 2017; Sherretts et al., 2017). There is extensive literature on what motivates offenders of conventional homicides, including on their consequences and costs (see e.g., DeLisi et al., 2010; Boduszek et al., 2012; Ryan et al., 2017), but less so about cannibalistic homicides.

The biology of cannibalism is a relatively young field of investigation and the phenomenon itself poses a challenge to evolutionary theory. Early ethologists (Lorenz et al., 2002) viewed cannibalism as a behavioral abnormality. Richard Dawkins predicted cannibalism to be rare, as for cannibalism there is too much danger of retaliation, which is less likely to be true in members of different species because of a built-in asymmetry (Dawkins, 1982). However, the dismissal of cannibalism is not justified in light of the empirical evidence (Polis, 1980). Numerous species show cannibalistic behaviors and most investigators today think that cannibalism is an adaptive behavioral strategy (Elgar and Crespi, 1992; Pfennig et al., 1994). Much like for the case of kin selection (Hamilton, 1964), one needs to explain how such destructive behavior can be evolutionary stable. In some specific conditions, cannibalizing conspecifics might become an evolutionary trait by providing a nutritional food source. Animals such as the Australian redback spider and the praying mantis profit from sexual cannibalism, when the female cannibalizes the male after copulation as a source of protein to increase the chance of survival for their offspring (Birkhead et al., 1988; Andrade, 1996). In any group where cannibalism occurs, whether it is for nutritional, cultural, or other reasons, cannibalism would seem to be evolutionarily profitable with higher likelihood if the offender does not cannibalize their own offspring. Therefore, an important theme in the study of cannibalism is whether the consumption of kin is avoided (Pfennig, 1997). For example, spadefoot toad tadpoles occur in two morphs, an omnivorous morph, which feeds on detritus and microorganisms, and a carnivorous morph, which feeds on shrimp as well as other tadpoles. Omnivorous morphs only rarely feed on tadpoles, but if so they feed indiscriminately, whereas the carnivorous morphs feed regularly on conspecifics and show a strong avoidance of kin. Indeed, occasionally carnivorous morphs have been seen to nip at kin tadpoles and spit them out (Pfennig et al., 1993). Similarly, in sticklebacks, a highly cannibalistic fish species, kin-avoidance and spitting out of conspecifics have been observed (Bruijn and Sevenster, 1982; FitzGerald and van Havre, 1987; Mehlis et al., 2008). These findings on animal cannibalism confirm theoretical predictions from kin selection theory (Hamilton, 1964). Hamilton’s theory attempts to resolve the important issue of the evolution of altruism and Hamilton’s rule describes the spread of an altruism-promoting allele. Furthermore, preventing cannibalism could also favor an organism’s own health as cannibalism is associated with a risk of parasite transmission, such as kuru (for a review see Liberski et al., 2019). Further, pathogens might affect cannibalistic behaviors. Specifically, there might be added pathogenic risks of eating kin, for example, because it might lead to infection with pathogens particularly dangerous for a specific genotype. However, there are not many studies on this topic. Studies on tiger salamander larvae suggest cannibalistic kin-avoidance, but there is also evidence for a cannibalistic kin-preference at least at some ages. A study that tested the role of pathogens in kin cannibalism in tiger salamander larvae was conducted by Garza and Waldman (2015) and provided some evidence for differential effects of pathogens transmitted by kin‐ and non-kin predation. Finally, cannibalism has been observed in prehistory (Villa, 2005), in 16th and 17th century Europe as medicinal cannibalism (Sugg, 2015), and in chimpanzees and bonobos, our closest relatives in the animal kingdom (Bygott, 1972; Goodall, 1977; Nishida and Kawanaka, 1985; Hamai et al., 1992; Fowler and Hohmann, 2010). As such, cannibalism continues to be a topic of scientific interest (Fouilloux et al., 2019). Lester et al. (2015) studied cannibalistic homicide and concluded that cannibals often have at least some mental problems. They focused on the psychological background of offenders and provide deeper insight in the psychology of such crimes (Lester et al., 2015). Another recent report (Raymond et al., 2019) also focuses on the psychological profile of cannibalistic offenders. These authors also agree that non-ritual and non-survival cannibalism is often pathological.

Here, we studied human cannibalistic homicide in modern societies. We defined a cannibalistic homicide as an act in which a human was killed and parts of the body were consumed or prepared for consumption. Conversely, we call a cannibalistic (homicide) offender a person who killed a victim and consumed parts of the body or secured parts of the body, including blood, for human consumption. As mentioned above, most work on cannibalistic homicides has so far focused on in depth psychological portrayals of individual offenders. However, we followed a different path as pioneered by Lester et al. (2015). These authors identified characteristics of cannibalistic offenders by comparing numerous cannibalistic offenders to non-cannibalistic serial killers. Like Lester et al. (2015), we pursued a statistical analysis of cannibalistic homicide. Police sources and press coverage facilitate obtaining detailed information about such cases. Indeed, many modern cannibalistic homicide cases have been described in depth in monographs. We obtained detailed data on cannibalistic homicides in multiple ways, including internet searches, scientific and (auto)biographical publication reviews, court records, and expert questioning. Such an internet-driven case search is effective but is subject to obvious sampling biases and reliance on “second hand” knowledge.

Our analysis was inspired by the seminal work of Daly and Wilson on Detroit homicides (Daly and Wilson, 1982) who showed that only a small fraction (6.3%) of conventional homicides has kin victims. We expected that cannibalistic homicides will have a different pattern from conventional homicides, with even less kin victims due to a biological repulsion and possibly pathogenic risk of eating kin. Until now, kin-cannibalism was examined in just one study on psychiatric patients with only five cases (Raymond et al., 2019). We hypothesized that the psychiatric illness of these patients may cause them to not be representative of all cannibalistic homicides. Here, we will compared cannibalistic homicides and conventional homicides in various countries, as reported in FBI data and in studies such as those by Daly and Wilson (1982) mentioned above, regarding offender and victim profiles, offender-victim relationship, and homicide methods. We furthermore explored patterns of the cannibalization itself.

## Materials and Methods

### Cases

We collected data from altogether 121 offenders from modern societies. We counted 631 victims (best estimate, with lower bound estimate of 542, and an upper bound estimate of 1,079 victims).

### Procedure

#### Our Approach

We assembled a list of cannibalistic homicides based on internet-searches, analysis of books and scientific publications, and expert questioning. Each avenue of investigation is described in detail below. As also extensively mentioned in the discussion, it is important to consider the general validity of such internet‐ and secondary source-based assessment of cannibalistic homicide. Such considerations are all the more important, because cannibalism is an emotionally charged topic and because research into cannibalistic practices has proven to be a source of controversy.

In our data set, we have used initials for all offenders and all victims. If only either first name or surname was known, we used only one initial. Some homicides were committed by multiple offenders. In these cases, we mention each offender individually in Supplementary Data Sheet 1, with the count of the homicides for the whole group. In Supplementary Data Sheet 2, we mention each victim once, and some of them will have multiple offenders listed. Thus, the total number of victims in Supplementary Data Sheet 1 is higher than Supplementary Data Sheet 2. The correct victim count is in Supplementary Data Sheet 2. In some cases, it is known that the offender had eaten or prepared meat from one or more bodies, but it is not entirely clear from which victim. Thus, in Supplementary Data Sheet 2, we have then noted that it is unknown for each victim from one offender whether or not that victim was cannibalized. In such cases, we know cannibalism, using our definition explained above, has occurred by that offender, but it is unclear for exactly which specific victim(s).

### A Fair Presentation of the Evidence

A major effort was made toward a fair presentation of the evidence:

1. Rating of evidence quality. We rated the quality of the evidence for each cannibalistic incident reported as poor (24%), good (35%), very good (20%), and excellent (20%). We provide these ratings to allow the reader an easy assessment of the data quality.
2. Keeping track of estimates. As already pointed out above, we relied in many instances on estimates about cannibalistic homicides. We developed a rating system for the quality of the sources, based on which we could establish a best estimate regarding victim count, in addition to the lowest and highest estimates for a number of victims of each offender (see Supplementary Data Sheet 1). For presentation purposes, we often had to pool established facts and estimates, but an effort was made to keep track of such estimates and provide this information in the respective figures and the respective tables.
3. Verification is easy. The very detailed data obtained here (offender initials, victim initials, dates, places, and murder methods) and presented in Supplementary Data Sheets S1 and S2 lend themselves for an easy verification/rejection of our claims.

### Exclusion Criteria

#### Cases Need to Qualify as Cannibalistic Homicide

Not all candidate cases of cannibalistic behaviors were included in our study. Specifically, we required that at least one case of cannibalistic homicide was established for each offender. Whenever possible we made this assessment based on conclusion by police investigator as communicated to the press and cited in the documentation available to us. Our study includes all homicides from a cannibalistic offender and not only those homicides for which the cannibalism was established.

#### Exclusion of Aboriginal Cannibalism

We focused on cannibalistic homicide in modern societies and excluded aboriginal cannibalism cases. The reason for such an exclusion is that the cultural mind set on cannibal practices might be quite different in modern and aboriginal societies. In modern societies, eating human meat is outlawed and consumption of pieces of a corpse is viewed as an act of mutilation and desecration. In the Fore from Papua New Guinea, however, a funerary cannibalism was practiced out of love and respect for the deceased (Whitfield et al., 2008). Hence, pooling such behaviors seems inappropriate.

#### Exclusion of Hunger Cannibalism

There are numerous cases of so-called hunger cannibalism, in which individuals chose to eat humans in order to escape starvation. While such cases of hunger cannibalism are very interesting (see our discussion), such extreme situations go along with drastically reduced behavioral options for offenders. Since our major interest relates to the behavioral choices of cannibalistic offenders, we chose to exclude these cases.

#### Exclusion of War Cannibalism

There are numerous cases of so-called war cannibalism, in which offenders had orders or permit to eat other humans, typically prisoners of war or subjugated people. Again, in the extreme war situation, the behavioral choices of cannibalistic offenders seem to be restricted and we chose to exclude such cases.

#### Exclusion of Merely Suspected Cases

When the cannibalistic homicide was not established with reasonable certainty, we excluded the cases (Supplementary Data Sheet 3). Prominent examples include German serial murder M.S., who removed numerous body parts of his victims, but whose cannibalism was not firmly established in the *post hoc* investigation after his death. Another excluded offender was American E.K., whose cannibalism confession was thought to be a tactical maneuver.

### Multiple Forms of Internet-Searches

The initial data set was assembled from multiple forms of internet-searches. These included:

*Searches for cannibalism and related keywords.*
*Searches from starter sets and crime related resources.* A key source in our initial establishment of our data set was the List of incidents of cannibalism.
*Searches for cross-links in net-sources and newspaper articles.* Once a cannibalistic incident was established, we carefully reviewed the corresponding internet coverage for cross-links to further cases.
*Searches for offender and victim names.* Searches for victim and offender names were routinely performed for all cases.
*Darknet, cannibal fora and the like.* We heard about such communication channels in the course of our research on cannibalism but avoided such links in their entirety.

### Literature Research, Books, and Papers

An extraordinary amount of publications deal with cases of cannibalistic homicides. Whenever we identified a monograph covering a cannibalism case, we ordered the respective book. Altogether, we screened 19 such monographs. We consulted four scientific publications describing original cannibalistic homicide cases (Rajs et al., 1998; Teixeira et al., 2012; Kapo et al., 2018; Raymond et al., 2019). We also obtained general literature (10 other books and papers) about cannibalistic homicides but did do so in a less systematic fashion. Last, we ordered 15 more books about the general topic of (serial/sexual) homicides for comparison data, as well as some mentions of cannibalism. A list of books consulted can be found in Supplementary Data Sheet 5.

### Original Data/Interviews

In a small fraction of cases, we also reviewed original evidence related to cannibalistic homicides such as interview transcripts, audio-visual interviews, court exhibits, and monuments commemorating victims. This source of information played only a minor role (for <5% of cases) in shaping the data set.

### Expert Questioning

We contacted 30 experts on forensic studies, criminology, or serial killers, in the USA, Germany, the Netherlands, and Denmark, to ask whether they had any additional cases or information to our data set. We got a reply from 13 experts, which revealed two additional cases, which were added to the data set.

### Literature

Additional literature consulted, other than those referenced to in the main text, is available in Supplementary Data Sheet 5.

### Instruments (Variable Coding)

#### Mental Health Score

To quantify mental health, we calculated a mental health score for each offender, ranging from 0 to 4. Scores were computed by adding +1 for each: prior-to-offense psychiatric institutionalization, report of auditory hallucinations, insanity (reduced responsibility) plea, and insanity (reduced responsibility) verdict. Scores for offenders who were unfit for trial because of mental health problems were computed similarly to as if they had received an insanity plea as well as an insanity verdict.

#### Individual Cases and Their Scoring

Our study on cannibalistic homicides focused on statistics of these crimes, such as: age and sex of offenders and victims, method of killing, details of the cannibalization, the relationship between victim and offender, and the mental state of the offender. Obtaining such statistics is not always obvious, as the available evidence may be lacking in various aspects. To highlight how we scored each case and used this scoring, we will first describe two individual cases. These examples will give the reader insight into the nature of the crimes, the characteristic of the evidence, and our scoring of the cases.

**Figure 1:**
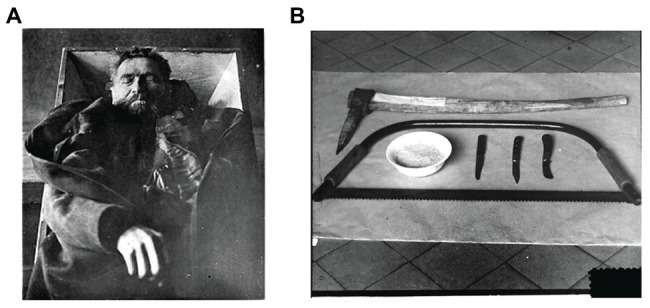
Profile and weapons of choice of cannibal K.D. **(A)** Headshot of K.D. in his grave (1924). This photo has originally been released by the police and is now available in the public domain. **(B)** Tools discovered in K.D.’s home, presumably used for his murders and later dismemberment of the bodies. Photo from the Medical Academy of Wrocław, presumably originally from the German Institute of Forensic Medicine in Breslau, is now available in the public domain.

K.D. (1860–1924; Figure 1A) was a German serial cannibalistic offender active at the beginning of the last century, committing his first known murder in 1903. He lived in Münsterberg (today Ziębice, Poland) and was caught when V.O., whom he invited into his home under the pretense of a free lunch, escaped his assault with an ax (Figure 1B). After V.O. fled K.D.’s home with a head injury, he accused K.D. V.O. was initially arrested for vagabondage, as K.D. was very well respected in Münsterberg. In the end, V.O. managed to convince the judge to have K.D. investigated. When the police came to K.D.’s home, they found large quantities of human meat in all kinds of buckets and various stages of preparation for human consumption. K.D. committed suicide the following night in jail and hence no confession or other first-hand knowledge about his deeds and motives are available. He kept notes, however, and noted the names of victims as well as their “slaughter weights”. Consequently 31 victims (5 women and 26 men) are known by name and a detailed police investigation uncovered remains of at least 42 humans in K.D.’s home. It seemed from K.D.’s notes and the police work on his case is that obtaining human meat was an important aspect of his crimes. Our scoring of the case was the following: We rated the quality of case documentation as excellent. We scored the number of 42 victims as an upper bound victim estimate; this is an unsatisfactory upper bound estimate, because it might well be too low, but no other evidence-based estimate was available. We scored 42 victims as the best victim number estimate and the 31 well-documented victims as a lower bound victim estimate. All victims were scored as probably cannibalized. Names, sex, and ages of the 31 known victims and the age of K.D. at the respective murders could also be extracted, while the remaining 11 victims were scored as of unknown sex and age. While it was not explicitly mentioned, we scored all victims as unrelated to K.D., because we find it implausible that in a well-documented case, such a kin relationship goes unmentioned; victim names support this reasoning. The relationship of K.D.’s murders to sexual acts was scored as unknown.

S.S.G. (born 1969), also referred to as “The Crossbow Cannibal,” was an English serial cannibalistic offender active during 2009–2010. He received a good education at a private grammar school and obtained a degree in psychology. In his youth, S.S.G. liked to shoot and dismember birds. In addition, at the ages of 17 and 23, he was convicted to prison sentences (3 and 2 years, respectively) for knife attacks. Later, he was diagnosed as a “schizoid psychopath.” He continued to pursue a PhD in homicide studies at Bradford University. In June 2009, at the age of 40, S.S.G. committed his first murder. He killed a 43-year-old sex-worker, S.R. Her body was never found. In April the next year, he struck again and murdered S.A. (aged 31), another local sex-worker. From S.A., only the shoulders, vertebrae, and connective tissue were found. One month later, he committed his third and last murder on sex-worker S.B. (aged 36). S.S.G. had also extensively dismembered her body: Police recovered 81 fragments of her corpse, and there must have been many more as the complete body was not found. Footage from private security cameras showed S.S.G. shooting S.B. twice with a crossbow, before turning around and posing for the camera. He claimed to have eaten from his victims, sometimes cooking parts of the meat, sometimes eating them raw. S.S.G. saw eating his victims as “part of the magic.” When charged, S.S.G. confessed to the three murders. He was sentenced to life imprisonment without a chance of ever being released. The press coverage of the case was extensive, and significant materials on the case were available to us, such as newspaper reports and a monograph on the case by journalist Cyril Dixon (2011). We scored the case as follows: we rated the quality of case documentation as excellent. We gave S.S.G. a mental score of 1 (on a scale from 0 to 4, where the higher score indicates lower mental health) due to his previous diagnosis of a “schizoid psychopath”. However, there are no claims that he had auditory hallucinations during the murders, he did not plea for insanity, and he was not sentenced to a psychiatric institution. We scored the number of three victims as an upper bound, lower bound, and best estimate. It is unclear which of the victims were cannibalized, so we left that unscored. S.S.G. had known the victims, at least briefly, so we scored the offender-victim relationships as unrelated acquaintances. We scored all three homicides as probably necrophilic/sex-related, based on video footage with one of the victims found on the offender’s computer.

### Statistical Analyses

A chi-square test of independence was performed for all comparisons.

## Results

### Cannibalistic Homicide Characteristics

**Figure 2:**
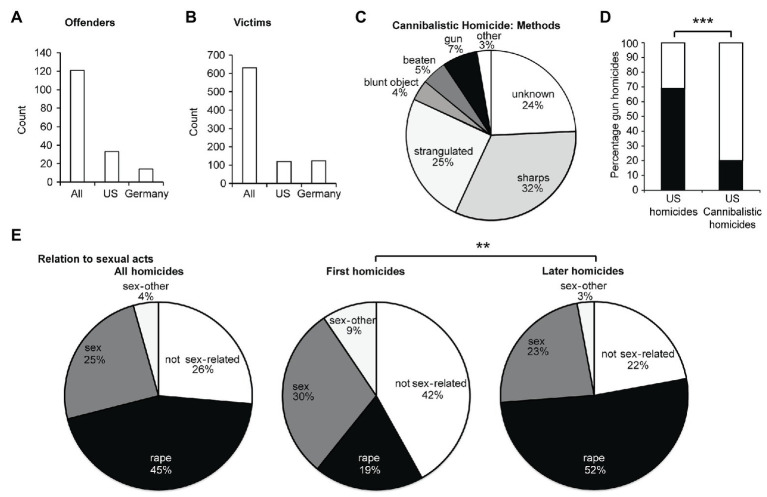
Rate, killing methods, and relation to sexual acts of cannibalistic homicides. **(A)** Offender numbers in our sample. **(B)** Victim numbers in our sample. All homicide victims of cannibalistic offenders are plotted, numbers refer to best estimates as defined in the methods. **(C)** Incidence of killing methods in cannibalistic homicide. **(D)** Fraction of US homicides and US cannibalistic homicides performed with guns (Fox, 1975). Conventional homicides in the US were more often performed with guns than cannibalistic homicides in the US [*X* ^2^(1) = 69.8, *p* < 0.001]. **(E)** Relation of cannibalistic homicides to sexual acts in all homicides by cannibalistic offenders (left), their first homicides (middle), and their later homicides (right). There was a significant difference between first and later homicides [*X* ^2^(3) = 9.4, *p* = 0.002].

An overview of our data set is given in Figures 2A,B. The full data are also provided in Supplementary Data Sheets S1 and S2. Cannibalistic offenders typically used manual methods to kill (stabbing, strangulating, and beating) rather than guns (Figure 2C). The murder methods observed were very different from conventional homicides in the US, most of which were carried out by guns (Fox and Zawitz, 2010; Figure 2D). Importantly, this difference in the method of killing also held up if we restricted the comparison to US cannibalistic offenders (Figure 2E). Many cannibalistic homicides were sex-related crimes (Figure 2E). Given the frequent nature of cannibalistic homicides with sexual acts, we also compared kin-avoidance in such cases to non-cannibalistic homicides with sexual acts. Sexual homicide accounts for 1.1–4% of homicides in which murder circumstances were known (Chan and Heide, 2009), which is much lower than the 74% of cannibalistic homicides of which such circumstances were known in our study (Figure 2E). For cannibalistic sexual homicides, the percentage of kin victims is only 0.8%. For non-cannibalistic sexual homicides in the United States 1976–2004, 2.9% of victims were kin (Fox, 2007). Another study of 77 convicted sex offenders of elderly victims (aged >60) reported a similar number: In 2.6% of cases, the offender was a family member (Burgess et al., 2007). Thus, even if we compared cannibalistic and non-cannibalistic homicides with sexual acts, the cannibalistic homicides had a tendency to have less kin victims [*X* ^2^(1) = 3.3, *p* = 0.068].

### Cannibalistic Homicide Offenders and Victims

**Figure 3:**
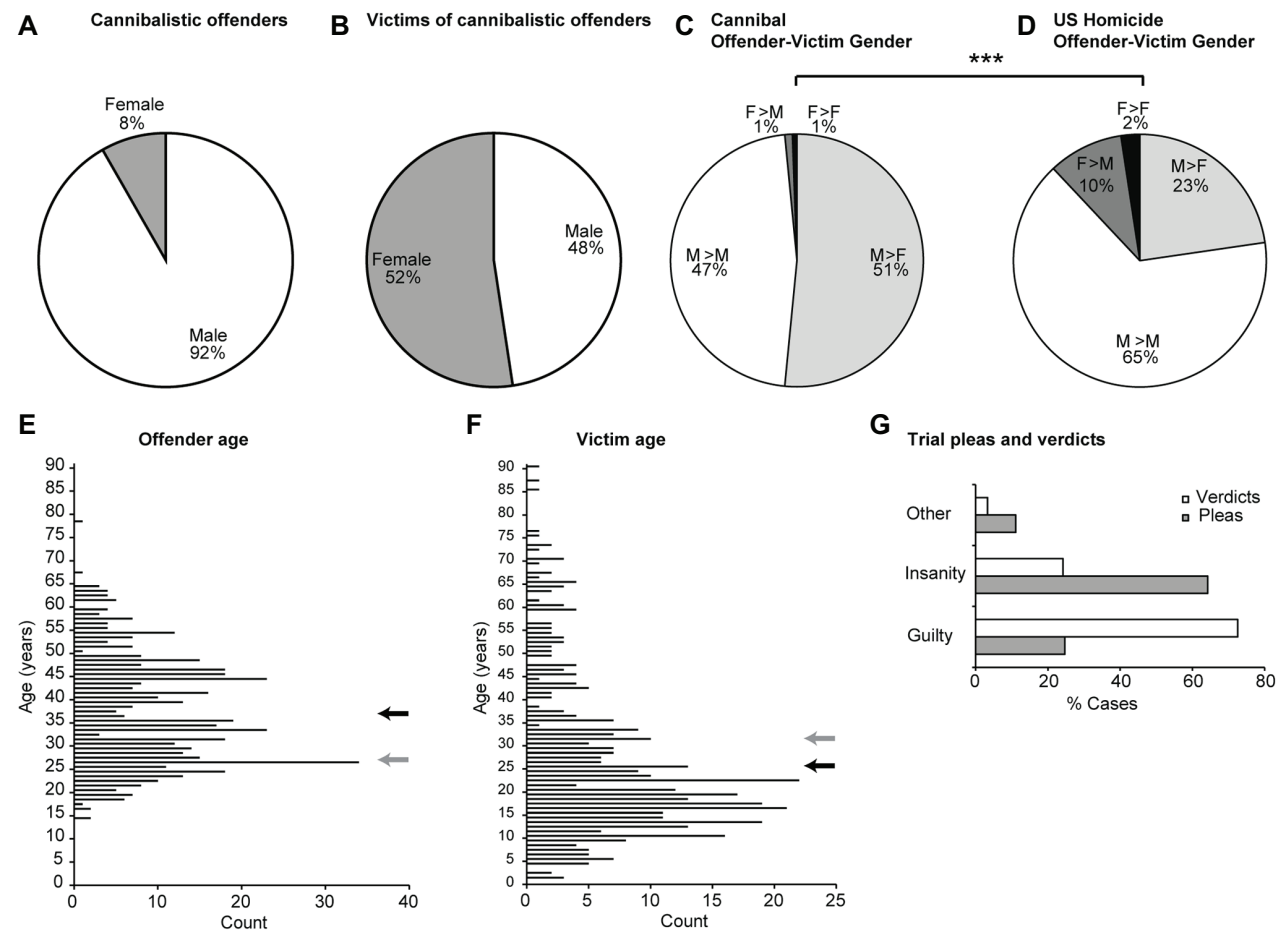
Gender and age of victims of cannibalistic homicides and of US homicides. **(A)** Gender of all cannibalistic offenders in this data set. **(B)** Gender of victims of all cannibalistic homicides in this data set. **(C)** Gender relationships in cannibalistic homicides. **(D)** Gender relationships in US homicides (Fox and Zawitz, 2010), which are different from the cannibalistic homicides in C [*X* ^2^(3) = 86.8, *p* < 0.001]. **(E)** Age of victims of all cannibalistic offenders in this data set. Black arrow indicates mean, gray arrow indicates mean age of US homicide offenders for comparison. **(F)** Age of victims of all cannibalistic homicides in this data set. Black arrow indicates mean, gray arrow indicates mean age of US homicide victims for comparison. **(G)** Pleas and verdicts in trials of all cannibalistic offenders.

Much like conventional homicide offenders all over the world, the vast majority of cannibalistic homicide offenders were men (Figure 3A). Cannibalistic homicide victims were roughly half men and women (Figure 3B). Male and female cannibalistic offenders targeted men and women equally (Figure 3C). Male victims were much more common in cannibalistic homicides than in conventional homicides in the US (Figure 3D). One of the surprising elements in our study relates to the age of cannibalistic homicide offenders (Figure 3E). Such offenders were old and there was a scarcity of offenders 17 or younger. This was strikingly different from US homicide offenders who were on average substantially younger (gray arrow), and many of whom were 17 years of age or younger. The average age of victims of cannibalistic homicide was only 25 years (Figure 3F). The victims of cannibalistic offenders were younger than US homicides victims (who were on average older, gray arrow). Next, we checked the pleas and verdicts during trials of these cannibalistic homicides. In line with what one might expect from the respective case reports, many cannibalistic offenders did plead insanity with considerable success in the courts (Figure 3G).

### Patterns of Cannibalization

**Figure 4:**
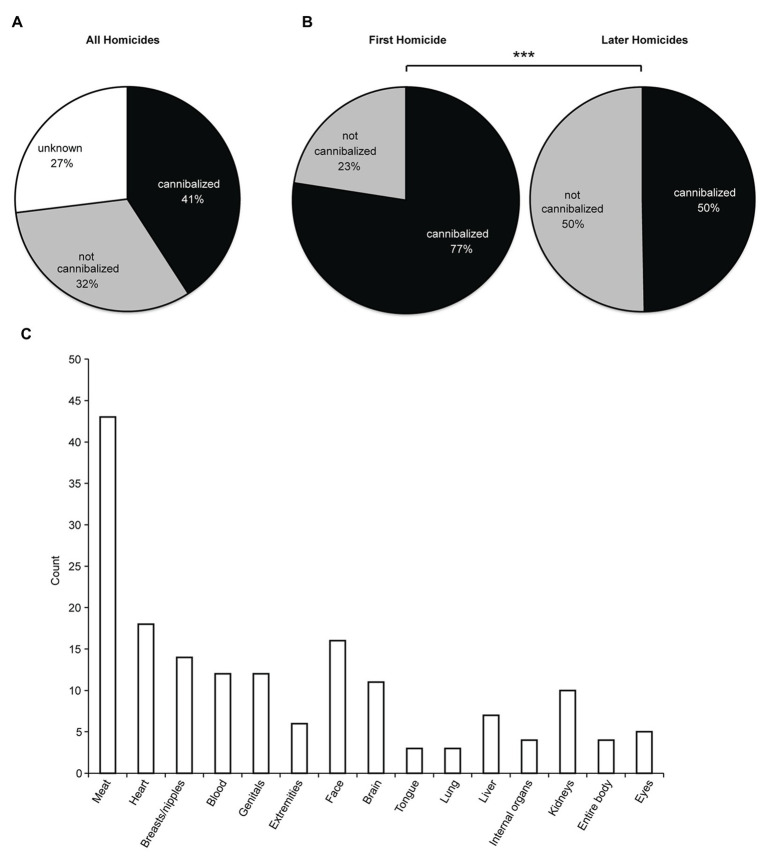
Patterns of cannibalization. **(A)** Cannibalization in all homicides of cannibalistic offenders. **(B)** Left, cannibalization in the first homicides of cannibalistic multiple offenders. Right, cannibalization in the later homicides of cannibalistic multiple offenders. There is a difference in cannibalization pattern between first and later homicides [*X* ^2^(1) = 24.2, *p* < 0.001]. **(C)** Cannibalized body parts. Each offender appears only one time or not at all in each reported category.

When we designed our study, we decided to include all homicides of cannibalistic offenders in our analysis. We made this decision because it is much easier to verify if a particular offender is cannibalistic than to verify if a particular homicide was cannibalistic. The distinction of cannibalistic and non-cannibalistic crimes is nonetheless important for our study. For comparisons about patterns of cannibalization, we only included the homicides for which information about cannibalization was available. As shown in Figure 4A, we could assign the cannibalization in homicide offenses to three categories: cannibalized, non-cannibalized, and cannibalization unknown. A potentially important difference was observed in the initial and later homicides of multiple cannibalistic offenders. Cannibalism was noticeably more common in their first crimes than their later homicides (Figure 4B). In Figure 4C, we give an account of the body parts cannibalized. This estimate was based on relatively incomplete information, because – as a result of desecration concerns – such details were not always reported on, or even relayed to the press. Still, it was evident that cannibalistic offenders most often went for the meat of their victims. This statistic was somewhat surprising to us, because in the reporting on cannibals genital/breast mutilations figured much more prominently than meat. Some cannibalistic offenders have reported that genitals were difficult to prepare/consume (A.M., interview; as well as A.F., his own letters; Fish and Borowski, 2014), and this might contribute to the fact that genitals were more often mutilated than consumed.

### Victim-Offender Relationships

**Figure 5:**
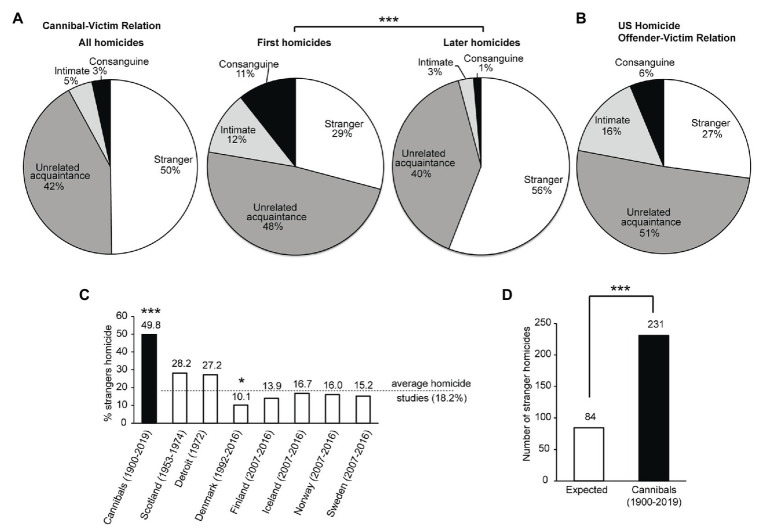
Offender-victim relationships of cannibalistic homicides and other homicides. **(A)** Offender-victim relationships in homicides of cannibalistic offenders, for all their homicides, only their first homicides, and the later homicides. There is a difference in offender-victim relationship between the first and later homicides [*X* ^2^(3) = 42.94, *p* < 0.001]. **(B)** Offender-victim relationships in all Detroit homicides (Daly and Wilson, 1982). **(C)** Fraction of homicides of strangers in cannibalistic offenses (this study) and other homicide studies (Gillies, 1976; Daly and Wilson, 1982; Lehti et al., 2019; Thomsen et al., 2019). The fraction of stranger homicides is different between cannibals and conventional homicides in other countries [*X* ^2^(7) = 52.2, *p* < 0.001, post-hoc pairwise comparison *X* ^2^ = 49.8, *p* < 0.001 for cannibalistic homicides and *X* ^2^ = 7.5, *p* = 0.03 for homicides in Denmark, others n.s., fdr adjusted]. See also Supplementary Data Sheet 4. **(D)** Expected and observed number of cannibalistic homicides on strangers, based on the average percentages from other homicide studies in C and the total number of cannibalistic homicides with a known offender-victim relationship in this study. The observed number of stranger victims in cannibalistic cases is lower than expected [*X* ^2^(1) = 321.4, *p* < 0.001].

We categorized offenders-victim relationships as either strangers, unrelated acquaintances, intimates, or kin. Complete strangers were defined as victims whom the offenders had never met until the moment of the homicide. Unrelated acquaintances included neighbors, friends, but also prostitutes they met a few hours before the homicide. Intimates were intimate partners, including (ex-)wives, and other romantic relationships. Kin were blood relatives only, excluding, for example, stepsiblings and foster children. Interestingly, cannibalistic offenders killed more strangers (Figures 5A–D) and fewer intimate partners (Figure 5A) than homicide offenders in Daly and Wilson’s study (Daly and Wilson, 1982) on Detroit homicide (Figure 5B). The fraction of victims who were strangers was much higher in cannibalistic homicides than in conventional homicides (Gillies, 1976; Daly and Wilson, 1982; Lehti et al., 2019; Thomsen et al., 2019) (Figure 5C, Supplementary Data Sheet 4). Based on the average percentage of stranger victims in conventional homicides in seven different areas, we expected a much lower number of strangers than we found to be the case for the victims in our sample (Figure 5D).

### Kin-Avoidance in Cannibalistic Homicide

**Figure 6:**
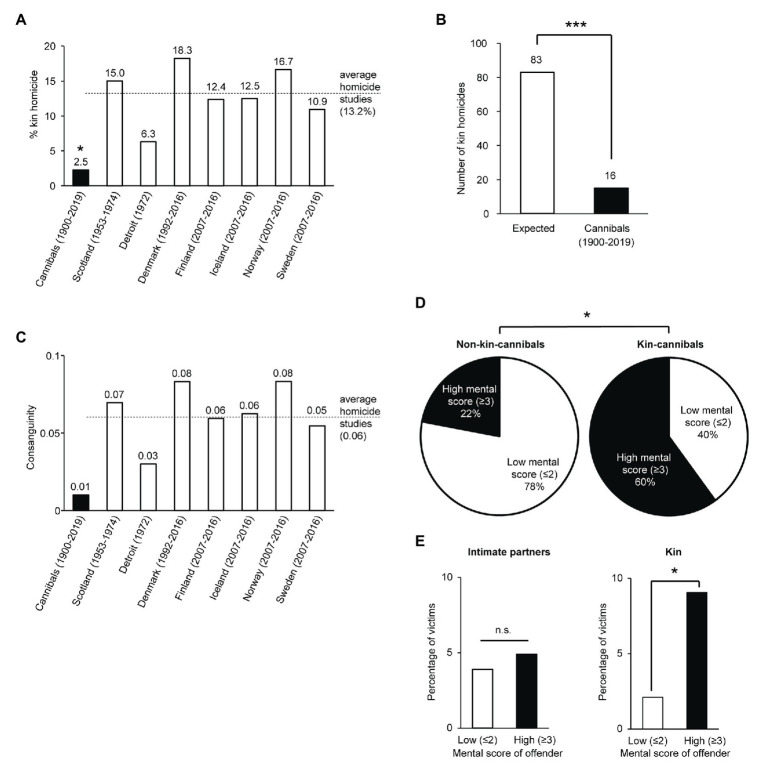
Kin-avoidance in cannibalistic and other homicides. **(A)** Fraction of kin homicides in cannibalistic offenses (this study) and other homicide studies (Gillies, 1976; Daly and Wilson, 1982; Lehti et al., 2019; Thomsen et al., 2019). The fraction of kin homicides is different between cannibals and conventional homicides in other countries [*X* ^2^(7) = 16.8, *p* = 0.02, post-hoc pairwise comparison *X* ^2^ = 8.7, *p* = 0.02 for cannibalistic homicides, others n.s., fdr adjusted]. **(B)** Expected and observed number of cannibalistic homicides on kin, based on the average percentages from other homicide studies in A and total number of homicides in this study. The observed number of kin victims in cannibalistic cases is lower than expected [*X* ^2^(1) = 62.3, *p* < 0.001]. **(C)** Consanguinity in cannibalistic offenses (this study) and other homicide studies (Gillies, 1976; Daly and Wilson, 1982; Lehti et al., 2019; Thomsen et al., 2019). **(D)** Mental health scores for non-kin (left) and kin-cannibals (right). Scores were computed by adding +1 for each: prior-to-offense psychiatric institutionalization, report of auditory hallucinations, insanity (reduced responsibility) plea, insanity (reduced responsibility) verdict. Offenders unfit for trial, because of mental health problems: +2. Contradictory or unclear reports were scored as 0.5 points. Low mental scores were considered 2 points or less, high mental scores were considered a score of 3 or 4. Kin cannibals had a high mental score more often than non-kin cannibals [*X* ^2^(1) = 4.5, *p* = 0.034]. **(E)** The percentage of victims which were intimate partners or which were kin for offenders with a low mental health score (≤2) and offenders with a high mental health score (≥3). Mean percentage of intimate partner victims for offenders with low mental health score: 3.9%, for offenders with high mental health score: 4.9%, *X* ^2^(1) = 0.009, *p* = 0.92. Mean percentage of kin victims for offenders with low mental health score: 2.1%, for offenders with high mental health score: 9.0%, [*X* ^2^(1) = 6.0, *p* = 0.01].

Even more interesting was the rarity (16 out of 631, 2.5%) of kin in the victims of cannibalistic offenders. This low incidence of kin attacks was markedly different from homicides in a variety of countries (Figure 6A), where the fraction of kin victims was between 6.3 and 18.3% (Gillies, 1976; Daly and Wilson, 1982; Lehti et al., 2019; Thomsen et al., 2019). The observed number of kin homicides in the sample of cannibalistic homicides was lower than expected based on the average percentage of kin victims in conventional homicide studies (Figure 6B). As expected, the consanguinity of cannibal-victim pairs was lower than consanguinity observed in conventional homicide studies (Figure 6C). Additional observations corroborate the idea of kin-avoidance in cannibalistic homicide. In 2 of the 15 cases, kin-cannibalism offenders spat out the remains of their kin. O.S. reported that she heard voices (the devil in her words) telling her to eat her son. She did not want to do it, however, and threw up when she did. P.R.F. emphasized in an interview that he chewed on his father’s heart, but did not eat it and spit it out. Spitting-out is less often reported in non-kin cannibalistic homicides (in 3 out of several hundred cases), presumably because eating human meat is part of the incentive for the crime. We also observed a noticeable difference in the state of mental health between kin‐ and non-kin offenders. The few kin-offenders had significantly more severe mental health problems (Figure 6D). Furthermore, we compared the percentage of victims who were intimate partners or who were kin for offenders with a low mental health score (≤2) and offenders with a high mental health score (≥3). The percentage of victims who were intimate partners of the offender was not different between offenders with low and high mental health scores (Figure 6E, left), while offenders with a high mental health score more often targeted kin than offenders with a low mental health score (Figure 6E, right). Collectively, our observations argue for powerful kin-avoidance mechanisms in cannibalistic homicide.

## Discussion

Cannibalistic homicides were very rare, often violent, manual and sex-related crimes. Victims were younger and offenders were older than in conventional homicide offenses. Cannibalistic offenders only rarely consumed kin and most who did suffered from serious mental problems.

Our data set consists of 121 offenders with approximately 631 victims. This is a very large number of victims, but note that we are dealing with the entirety of easily accessible cannibalism cases in modern societies since 1900. The case numbers in the US and Germany might be of particular relevance, because, in these two countries, we made a special attempt for a complete coverage of cases. Unsurprisingly, cannibalistic homicide is an exceedingly rare crime, accounting for a minute fraction of homicides. For the US in the period 1960–2018, we estimated the fraction of cannibalistic homicides, being the number of cannibalistic homicides divided by the total homicides [Uniform Crime Reporting (UCR) Program Federal Bureau of Investigation, 2020], to be 0.01%.

The cannibalistic homicides described here had many hundreds of victims but included only one cannibalized neonate. The two other children 2 years or younger killed by cannibalistic offenders were not cannibalized. Since in non-cannibalistic homicides the killing of children 2 years or younger is quite common (accounting for ~2.6% of homicides as reported in FBI data), it appears that this victim population is largely missing in cannibalistic offenses, i.e., we would have expected >15 cases. From a biological perspective, the absence of cannibalistic neonaticide in humans is surprising. Human neonaticide shares many features of cannibalistic neonaticide in rodents and lagomorphs (Sawin et al., 1960; DeSantis and Schmaltz, 1984). In humans, neonaticide, as defined by the killing of a neonate on the day of its birth by his/her own mother, incidence varied from 0.07 to 8.5 per 100,000 births (Tanaka et al., 2017). This behavioral pattern is strongly promoted by maternal stress and intrusion by novel partners or detection of predators. Thus, even though neonaticide has been suggested to be a prototypical “biologically” predetermined behavior (Hausfater and Hrdy, 2017), it still shows differences between rodents and humans.

Our data suggest kin-avoidance in cannibalistic homicide. About 97.5% of the victims were non-kin, a statistical difference to occurrence of kin-homicides in conventional homicides (Figure 6). The consanguinity of victim-offender pairs was very low. This conclusion agrees with findings from two other large-scale studies on cannibalistic homicides (Rajs et al., 1998; Lester et al., 2015). A diverging result with a large fraction of kin-cannibalism was only observed in one study with only five cases (Raymond et al., 2019). We think these discrepancies reflect different sampling of cases, i.e., while our internet search sampled mostly cases by news coverage, Raymond et al. (2019) focused on psychiatric patients. Indeed, in our sample the cannibalistic offenders with the most obvious mental problems killed more kin (Figure 6D). This reduced kin avoidance in offenders with mental problems might not be limited to cannibalistic homicides. Offenders of conventional homicides in Scotland targeted three times more kin when they had a psychiatric diagnosis at the time of trial. The fraction of kin victims for offenders with a psychiatric diagnosis at the time of trial was 30.9%, compared to a kin victim rate of 11.3% for mentally healthy offenders (Gillies, 1976).

The fact that mental problems can reduce kin-selectivity in cannibalistic homicides does not argue against the existence of strong anti-kin-ingestion mechanisms, whether such mechanisms are a result of nature, nurture, or a combination of both. In rats, there are kin-responsive neurons in the lateral septum (Clemens et al., 2020), which might point to a possible biological basis of kin recognition at least in rodents. Humans use stable psychosocial cues to distinguish different types of kin from non-kin, and the cues used for kin detection depends on the specific dyad (Lieberman et al., 2007; Tal and Lieberman, 2008; Antfolk et al., 2018; Billingsley et al., 2018). One such cue is early co-residence between purported siblings as suggested by Westermarck (i.e., the hypothesis of Westermarck, 1922). It might be that this system of kin detection is affected in offenders with a psychiatric diagnosis and thereby prevents the offenders from recognizing and avoiding kin.

Spitting out conspecifics or parts of them is a characteristic behavior of cannibalistic animals and humans. We found five such throwing up /spitting out events, which may not sound like a lot in 631 victim cases, but our documentation is not comprehensive and it is important to keep in mind that many humans may eat a thousand meals a year without any one such throw up event. In sticklebacks, a strongly cannibalistic fish species, spitting out of newly hatched fish has been carefully documented and referred to as “testing” (Bruijn and Sevenster, 1982). Similarly, the cannibalistic spadefoot tadpoles have been seen to nip at and spit out conspecifics (Pfennig et al., 1993), a behavior referred to as “tasting”. Our observations on humans and a review of the animal literature suggest, however, that it is very unlikely that spitting out meat from cannibalistic events indeed reflects a sensory discriminatory behavior or gustatory “tasting”. The reasons, why we reject the tasting/testing hypothesis are the following: (i) Stickleback, spadefoot tadpoles, and humans have potent non-gustatory kin-discrimination mechanisms. In stickleback (Mehlis et al., 2008) and in salamander tadpoles (Pfennig et al., 1994), such mechanisms are olfactory in nature and in humans and apes (Parr and de Waal, 1999) such mechanisms are presumably visual; (ii) when tested intadpoles by nare occlusion, olfactory mechanisms were necessary for kin discrimination, whereas the remaining gustatory mechanism (after nare occlusion) were insufficient for kin-discrimination (Pfennig et al., 1994); (iii) human gustatory kin-discrimination appears highly implausible from our screening of cannibalism reports. On numerous occasions, human meat was sold (and probably eaten) by cannibals as ostrich, pork, horse, or tenderloin, mostly without any customer complaints reported. For instance, J.R.M. sold meat of his victims as special barbecue meat in a stand next to his trailer. He mixed the meat together with pork, which he claims tastes very similar to human meat, so that nobody could tell the difference (Serial killer who “cut up victims and sold them as BBQ” dies, 2017). If humans cannot discriminate human meat from beef, how could humans or animals taste kin? (iv) “Testing” behavior in stickleback is strongly dependent on the state of satiation (Bruijn and Sevenster, 1982); it is not obvious why a sensory discriminatory behavior should strongly depend on the state of satiation; and (v) in interviews with human cannibalistic offenders, they do not report that kin had a bad taste. We propose an alternative explanation for cannibalistic spitting-out: We suggest that this behavior is driven by internally generated repulsion and reflects a conflict between kin-protective, anti-cannibalistic drives, and predatory/consumptive systems. This explanation makes sense, because (i) we do not assume gustatory kin-discrimination, (ii) it is consistent with strong dependence on internal variables like hunger, and (iii) it fits with reports from cannibalistic offenders. In our explanation we explain a contradictory behavior (spitting out, a reversion of the decision to ingest), by an internal contradiction (preying vs. protection of potential kin).

Forensic awareness could be another explanation for the kin-avoidance observed in our study. Accordingly, cannibalistic offenders would avoid eating kin in order to escape prosecution, which is conceivably more likely for kin offenses. While we think forensic awareness is an important consideration, we do not think this hypothesis can fully explain our data. In particular, we do not see any evidence for a differential “forensic awareness” of cannibalistic and conventional offenders. Instead, many cannibalistic offenders enjoyed the celebrity emanating from their deeds; indeed, cannibal P.K. sends a letter to the press detailing the whereabouts of the grave of one of his victims, an action not speaking to forensic awareness. Also, other considerations do not align with the forensic awareness hypothesis. Offenders of cannibalistic crimes who showed forensic awareness were excluded from this study. For example, it is thought that the “confession” of cannibalism by E.K. is the result of forensic awareness, and he is, therefore, excluded from this study on the grounds of not enough evidence for cannibalism (Supplementary Data Sheet 3). However, we cannot exclude that other offenders still included in our study had a similar tactic unknown to us. It could be that offenders with forensic awareness will target strangers more often than family to dismiss any suspicions which might fall on them. In that case, one might argue that a reduced mental health also likely reduces the forensic awareness and that that could explain our finding that offenders with reduced mental health targeted kin more often. While we can not exclude this theory, we deem it unlikely because (i) this would then also be true for non-kin relatives such as intimate partners, and this is not the case (Figure 6E), and (ii) we excluded cases with suspicions of forensic awareness, and in first-hand reports by cannibals included in this study, they claim to really kill and eat for the purpose of pleasure, and (iii) cannibalistic offenders, who had enough forensic awareness to never be detected, are also not included in this study for the obvious reason that they are unknown. According to the theory above, uncaught cannibalistic offenders would target strangers more often than family, and those cannibals are missing from our study. Thus, if this is true, we are missing more strangers victims but we are not missing as many kin victims, in which case the actual effect size would be even larger than reported here.

War and hunger related cases of cannibalism appear to share features of cannibalistic homicide identified here. In war crimes, it is often the enemy which is cannibalized (Tanaka, 2018). In hunger-related cannibalism there are also indications of kin-avoidance. In the famous Uruguayan Air Force flight 571 incident, where starving victims of the plane crash fed on meat from deceased co-passengers to survive, it is said that at least some survivors made efforts to avoid eating kin (Arijón, 2008). Specifically, when one of them could only eat his kin for survival, he decided to cross the Andes to search for help instead (Parrado and Rause, 2013), which is the journey which eventually led to their rescue. Internally generated disgust is evident in such cases, i.e., the plane crash victims were appalled by eating human meat.

To achieve a correct interpretation of the results reported here, it is necessary to consider the limitations of our data set. Cannibalistic homicide is a secretive crime and our study is based on second hand and potentially distorted information. Thus, despite the best of our efforts, some errors are inevitable and we expect that our data set may contain mistakes about homicides, offenders, victims, and victim-offender relationships. The case of cannibalistic offender J.K. provides a warning. No less than five putatively innocent people were arrested for his crimes (three of whom committed suicide and a fourth was wrongly convicted of murder). Furthermore, some homicide offenders may give a calculated “confession” of cannibalism in order to use an insanity plea, as is suspected to be the case with E.K. (who for this reason has been excluded from this study, see Supplementary Data Sheet 3). In anthropology, the mere existence of human cannibalism in, for example, aboriginal cultures has been questioned on grounds of sensationalistic reporting in travelogs (Arens, 2004). Does this mean that one can dismiss our evidence about cannibalistic homicides, because these crimes seldom have eyewitnesses or video evidence and, without exception, incidents have been hyped by the press? We think the answer is a resounding no. The hundreds of cases documented in our report leave no room for doubt. We acknowledge, however, that our worldwide collection of cases is subject to a variety of sampling biases.

### Limitations of Our Approach

The value of the data provided in our study might be limited by the following weaknesses:

1. The secretive nature of cannibalistic homicide. Cannibalistic homicide is nothing that can be openly performed in modern societies. The vast majority of cannibalistic homicides described here have been performed secretively and often there have been considerable efforts by offenders to destroy evidence. Hence, this paper largely describes deeds that nobody witnessed and cannibalism – the defining characteristic of what is talked about here – has rarely been directly established.
2. The need for estimates. Given the secretive nature of cannibalistic homicide, many details of the offenses described here can only be estimated. Many of these estimates are based on excellent evidence such as confessions, post-mortem examinations, or strongly suggestive circumstance (a child’s hand cooking in salted water on the stove), but nevertheless the evidence remains inferential.
3. Sampling biases. Perhaps the biggest problem of the data presented here is that our “news-based” search for cannibalistic incidents is subject to sampling biases. In particular, the data presented here will be biased towards particularly newsworthy cases with high victim numbers or gruesome case details.
4. Outright distortions. Sensationalism in the press might also lead to inflated presentation of evidence and distortion of the facts.
5. Reliance on second hand information. Our data set relies largely on web and newspaper reports and less so on official documents (original verdicts, interviews, letters and notes from the cannibal offender, autobiographies by offenders, and the like). Hence, verification of incident details is largely indirect.
6. Unintentional mistakes. Our data set is very large and unquestionably contains mistakes. Such mistakes would happen less in official documents double-checked and based on primary crime evidence but are unavoidable in our type of analysis.
7. Language barriers. We decided for a worldwide search of cannibalism cases, which allowed us amassing a large sample, but led us to encounter language problems. In several instances we used tools such as Google Translate to check local news sources. Such tools are powerful, but imperfect.

### Strengths of Our Approach

1. Numbers. The biggest strength of the worldwide internet-based search for cannibalistic homicides is the sheer number of cases it returns.
2. Substantial coverage. The news coverage of cannibalistic cases is substantial. In a contemporary society, it is therefore simply unlikely that a cannibalistic homicide is not covered, unless (a specific detail of) a case is prohibited to be covered by the press.
3. Rich detail. Our data set contains very detailed information about cannibalistic homicides.
4. Good estimates. A lot of the evidence presented here is inferential, but many of the estimates presented here are good estimates. In modern societies an immense amount of effort is made to clear up homicides. Thus, a lot of the estimates about the deeds of serial murderers presented come from police investigators, who spent years of their life chasing the perpetrators; such detailed police work entitles to estimates about the deeds of offenders.
5. Documentation effort in internet-data sets. A lot of our searches and results rely on pages like murderpedia.org and lists of cannibalistic incidents. Many of these data sets do not comply with strict scientific standards of referencing; nevertheless, it would be a mistake to underestimate the effort and expertise that went into aggregating these data sets.
6. Access to rare cases. Cannibalistic homicides are rare and if one wants to investigate subclasses of such cases (i.e., very rare cases) the comprehensive search strategy is indispensable.
7. Informal cross validation. Whenever possible we informally cross-validated data sets against each other.

Our results indicate that cannibalistic homicide is a distinctive offense with a special pattern of murder methods, a strong relation to sexual acts, distinctive patterns of victims and offenders, and unique victim-offender relationships. There is a seemingly high amount of kin-avoidance in such crimes, in particular, if offenders do not suffer from serious mental health problems. We suggest that kin-avoidance and spitting out of conspecifics might be triggered by internally generated disgust against kin-ingestion.

## Data Availability Statement

All relevant data are contained within the article/Supplementary Material, as well as on a dedicated website: cannibalismresearch.org.

## Author Contributions

MB: conceptualization. MO and MB: methodology. MO and MB: investigation. MO and MB: formal analysis. MO and MB: visualization. MO and MB: writing. MB: supervision. MO and MB: funding acquisition. All authors contributed to the article and approved the submitted version.

### Conflict of Interest

The authors declare that the research was conducted in the absence of any commercial or financial relationships that could be construed as a potential conflict of interest.

## Supplementary Material

Supplementary Data Sheet 1 List of the 121 cannibalistic offenders used in this study. This list is not meant as a complete overview of cannibalistic offenders worldwide during this period. Rather, it reflects the results from our limited search based on newspapers, books, and internet sources.

Supplementary Data Sheet 2 List of the 631 victims from 121 cannibalistic offenders used in this study. This list is not meant as a complete overview of victims from cannibalistic offenders worldwide during this period. Rather, it reflects the results from our limited search based on newspapers, books, and internet sources.

Supplementary Data Sheet 3 Cannibalistic offenders excluded from our data set.

Supplementary Data Sheet 4 Relationship between offender and victim in conventional homicides (Gillies, 1976; Daly and Wilson, 1982; Lehti et al., 2019; Thomsen et al., 2019) and cannibalistic homicides (this study).

Supplementary Data Sheet 5 Literature consulted during the creation of the data set.
